# Anastomotic leak test using indocyanine green during laparoscopic Roux-en-Y gastric bypass: A cohort study

**DOI:** 10.1016/j.amsu.2022.104939

**Published:** 2022-11-17

**Authors:** Giovanna Pavone, Alberto Fersini, Mario Pacilli, Pasquale Cianci, Antonio Ambrosi, Nicola Tartaglia

**Affiliations:** a Department of Medical and Surgical Sciences, University of Foggia, Viale Pinto, 71122, Foggia, Italy; b General Surgery Unit, Bonomo Hospital, Viale Istria, 76123, Andria, BT, Italy

**Keywords:** Laparoscopic Roux-en-Y Gastric Bypass, Indocyanine green test, Anastomotic fistula, Gastro-jejunal leak

## Abstract

**Background:**

Indocyanine green (ICG) can be injected into the human bloodstream and it allows us to show stomach vascularity in real time. The aim of our study is to observe the preliminary results of the application of indocyanine green fluorescence (IGF) during laparoscopic Roux-en-Y Gastric Bypass (RYGB in our center and how the perfusion of the gastro-jejunal anastomosis affects the onset of fistula.

**Materials and methods:**

30 consecutive patients underwent RYGB with ICG fluorescence angiography at our center from January 2020 to December 2021.5 ml of ICG were then injected intravenously to identify the blood supply of the stomach and the gastro-jejunal anastomosis. The UIN for ClinicalTrial.gov Protocol Registration and Results System is: NCT05476159 for the Organization UFoggia.

**Results:**

In the RYGB tested with ICG, we all have adequate perfusion but despite this a methylene blue test was positive and allowed us to reinforce the suture of the gastro-jejunal anastomosis.

**Conclusion:**

Intraoperative ICG testing during laparoscopic RYGB may be helpful in determining which patients are at an increased risk for leakage but multiple factors concur to the pathophysiology and the incidence of gastric fistula not only the perfusion.

## Highlights

• Gastric Bypass is a common operation for weight loss in patients with severe obesity.
• In regards to Roux-en-Y gastric bypass (RYGB), anastomotic leaks are their most common complications with an incidence of 0–8%.
• Indocyanine green fluorescence angiography (ICG) is an emerging technology that has been used in an attempt to reduce the incidence of anastomotic leaks.

## 1 Introduction

Morbid obesity has become a major global health threat that leads to severe morbidity including diabetes, hypertension, obstructive sleep apnea, degenerative joint dis-ease and cardiovascular diseases [1].

Bariatric surgery is recognized as the most effective treatment for morbid obesity, maintaining a stable weight reduction in the long term and reducing comorbidities, with a favorable impact on mortality [[2], [3], [4]].

Gastric Bypass is a common operation for weight loss in patients with severe obesity. The procedure was developed in the 1960s by Drs. Mason and Ito who observed significant weight loss in a patient undergoing partial gastrectomy for peptic ulcer disease.

As expected, any increase in the frequency of a procedure usually unveils a significant number of related complications. Most of the complications of bariatric surgery are usually observed in the postoperative period.

Ninety-day mortality is very low (less than 0.5%). The morbidity of the procedure is classified into early complications and late complications [5].

Early complications (0–30 days):

• Venous thromboembolism (VTE)
• Anastomotic leak
• Infection
• Intestinal blockage
• Stenosis Gastro-Jejunal

Late complications.

• Intestinal blockage
• Dumping syndrome
• Marginal ulcer
• Gastrointestinal fistula
• Gallstones
• Incisional hernia
• Nutritional deficiencies

More information on complications after gastrectomy is beyond the scope of this review article. These are very complex pathological processes with specific etiologies, pathophysiology and treatments.

Gastric fistulas are the most common complication observed after gastric sleeve surgery. A meta-analysis of almost 10,000 sleeve gastrectomies conducted by Parikh et al. revealed that 2.2% of these procedures were posteriorly complicated by fistula development. In regards to Roux-en-Y gastric bypass (RYGB), anastomotic leaks are their most common complications with an incidence of 0–8% [6,7].

Indocyanine green fluorescence angiography (ICG) is an emerging technology that has been used in an attempt to reduce the incidence of anastomotic leaks. ICG is a water-soluble tricarbocyanine dye that remains in the intravascular compartment until excretion and has a plasma half-life of between 3 and 5 min [8]. Importantly, the ICG absorbs light at a wavelength of excitation between 750 and 800 nm while emitting light at emission wavelengths greater than 830 nm or more [9]. Using near-infrared imaging systems, vascular perfusion to the gastro-jejunal anastomosis can be assessed intraoperatively, allowing surgeons to anastomose a well perfused bowel or to reshape a poorly perfused anastomosis. 5 ml of ICG were then injected intravenously to identify the blood supply of the stomach and the gastro-jejunal anastomosis.

The aim of our study is to observe the preliminary results of the application of indocyanine green fluorescence (IGF) during laparoscopic Roux-en-Y Gastric Bypass (RYGB) in our center and how the perfusion of the gastro-jejunal anastomosis affects the onset of fistula.

## 2 Materials and Methods

We conducted a cohort study with 30 consecutive patients underwent RYGB with ICG fluorescence angiography at our center from January 2020 to December 2021.

During the period of interest, 10 male and 20 female patients underwent RYGB by the same surgical team with the same standardized technique.

The mean age was 43.6 in the male group and 37.6 in the female group (mean 39.18).

The mean preoperative BMI was roughly the same in the two groups (45.16 in the male group vs 45.06) (in the female group).

At least one major comorbidity was found in all patients: the most represented was hypertension (19 patients, 63.33%), followed by diabetes (6 patients, 20%), COPD and/or OSAS (4 patients, 12, 3%) and osteoarthritis (1 patient, 3.33%).

### 2.1 Five patients had previous sleeve gastrectomy surgeries

Adequate perfusion was defined as “the direct and clear visualization of the fluorescence along the gastric tube and the anastomosis, relative to the excised specimen, after an estimated time of 150–180 s after i.v. Administration injection "(Fig. 1a, Fig. 1ba–b).

**Fig. 1a:**
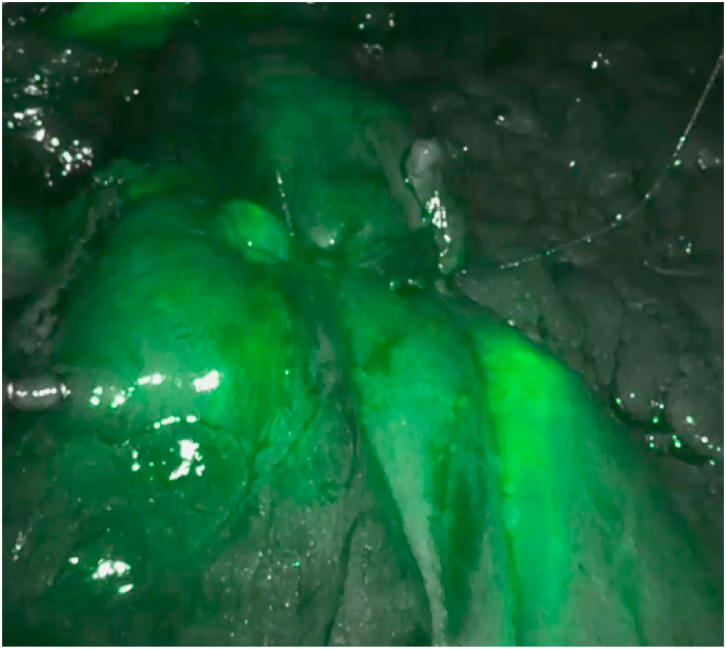
Evaluation of perfusion of gastro-jejunal by using Indocyanine green test. (For interpretation of the references to colour in this figure legend, the reader is referred to the Web version of this article.)

**Fig. 1b:**
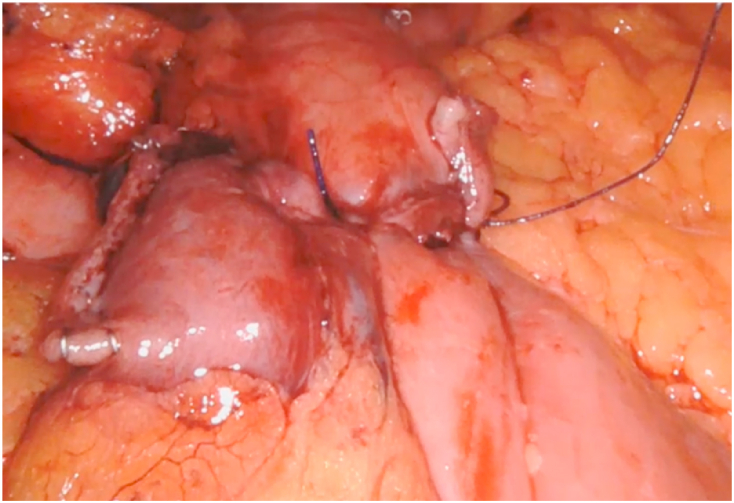
Intraoperative picture of the gastro-jejunal anastomosis.

In case of inadequate perfusion, our expected options were binding reinforcement with fibrin glue or with sutures.

A methylene blue test is routinely performed after fluorescence.

The procedure ended with the placement of an intra-abdominal drain along the suture line.

On the second postoperative day, a routine swallow test with Gastrografin is performed.

After the Gastrografin swallow test, if negative, patients were given a liquid diet for 1 day, then a semi-liquid diet and were discharged on the third postoperative day.

Our follow-up was performed routinely with blood tests and clinical examination at 3-6-12 months. The UIN for ClinicalTrial.gov Protocol Registration and Results System is: NCT05476159 for the Organization UFoggia (https://clinicaltrials.gov/ct2/show/NCT05476159).

This work is fully compliant with the STROCSS criteria [10].

### 2.2 Eligibility criteria

Adult patients of both genders with morbid obesity defined as BMI>40 kg/m^2^or BMI>35 kg/m^2^with at least one associated major comorbidity were included. We excluded patients with secondary obesity due to endocrine and psychological disorders and patients unwilling to comply with postoperative diet and exercise program.

## 3 Results

The procedure was performed in all patients without ICG-related adverse events.

The mean operative time was 123 min. No conversion to laparotomy was performed.

The blood supply was rated “satisfactory and adequate” in 29 patients, only 1 patient showed ischemic areas at the anastomosis, confirmed by intraoperative methylene blue test positive, a suture reinforcement of the gastro-jejunal anastomosis was performed with Vycril stitches.

Routine swallowing of Gastrografin on the second postoperative day was negative for leaks in all patients.

No patient showed signs or symptoms of leaks at the gastro-jejunal anastomosis.

Analyzing our database, we performed 26 RYGBs without ICG tests from 2017 to 2019; the total number of leakage was 0 and our leakage rate was 0%.

In the RYGB tested with ICG, we have only 1 case of inadequate anastomotic perfusion with a positive methylene blue test and allowed us to reinforce the suture of the gastro-jejunal anastomosis (Fig. 1a, Fig. 1ba - b).

## 4 Discussion

Anastomotic leaks following RYGB can be caused by a number of factors including those compromising healing such as mechanical tension and ischemia [[11], [12], [13], [14]] but are also associated with other factors such as BMI, age and the postoperative course [15].

The reported incidence of gastro-jejunal leakages after LRYGB has been reported to range between 1 and 3% [[16], [17], [18], [19]].

The diagnosis of gastrointestinal leak after bariatric surgery can be challenging. The patient's presentation varies according to the type and timing of the leak and the patient's systemic inflammatory response. Patients with morbid obesity may show uncertain presentations, leading to late diagnosis and potentially catastrophic consequences.

There is still uncertainty about the pathophysiology of anastomotic leak after RYGB: the cause may vary from person to person. It may be caused by a problem with the tool or materials used to close the anastomosis during surgery. It may be because of problems with the blood flow in the area after surgery. Or it may be linked to other reasons for poor wound healing, such as diabetes or smoking [[20], [21], [22], [23]].

The use of routine intraoperative methylene blue leak testing is still discussed as it may be useful for identifying suture line rupture but not for identifying areas at increased risk of subsequent leakage [24].

Recently, much interest has been focused on the possibility of assessing blood supply during surgery with the use of ICG fluorescence angiography, which is a real-time, inexpensive and feasible method of establishing vascularity in an area [25]. It has been fully and positively approved in most laparoscopic procedures [26] but there is still little literature on its usefulness in the bariatric procedure.

Therefore, our aim was to evaluate whether intraoperative ICG fluorescence angiography could lead to estimating the ischemic area [27,28].

In our study we tested 30 patients undergoing RYGB with ICG tests and only 1 patient showed an ischemic area at the anastomosis confirmed by the methylene blue test, therefore we considered it necessary to reinforce with Vycril points. This solution in this case proved to be sufficient to avoid the anastomotic leak.

Despite this, the cause of the anastomotic leak during RYGB is multifactorial, therefore not only the use of the ICG test can be a valid tool to avoid the leak caused by ischemia. A limitation of this study is the low number of patients.

## 5 Conclusions

We can conclude that ICG test in our study proved to be a valid tool to identify a possible ischemic area of the anastomosis confirmed by a positive methylene blue test, this forced us to reinforce the anastomosis. But multiple factors contribute to the pathophysiology and incidence of anastomotic fistula in the context of the RYGB operation. Therefore, intraoperative testing of the ICG can be helpful in determining which patients are at greater risk for anastomotic leak and whether additional measurement is needed during surgery, but further studies are needed to determine if the ICG test predicts leak due to the ischemia.

## Provenance and peer review

Not commissioned, externally peer-reviewed.

## Ethical approval

The ethics committee of our institution approved the study.

## Sources of funding

GIOVANNA PAVONE, ALBERTO FERSINI, MARIO PACILLI, PASQUALE CIANCI, ANTONIO AMBROSI, NICOLA TARTAGLIA. declare haven't been funded.

## Author contribution

(I) Conception and design: GIOVANNA PAVONE.

(II) Administrative support: ANTONIO AMBROSI.

(III) Provision of study materials or patients: ALBERTO FERSINI.

(IV) Collection and assembly of data: PASQUALE CIANCI.

(V) Data analysis and interpretation: MARIO PACILLI.

(VI) Manuscript writing: All authors.

(VII) Final approval of manuscript: NICOLA TARTAGLIA.

## Registration of research studies

Name of the registry: ClinicalTrials.gov
Unique Identifying number or registration ID: NCT05476159
Hyperlink to your specific registration (must be publicly accessible and will be checked): https://clinicaltrials.gov/ct2/show/NCT05476159

## Consent

Informed consent was obtained from all individual participants included in the study.

## Guarantor

The Guarantors are Professor Nicola Tartaglia and Professor Antonio Ambrosi.

## Declaration of competing interest

GIOVANNA PAVONE, ALBERTO FERSINI, MARIO PACILLI, PASQUALE CIANCI, ANTONIO AMBROSI, NICOLA TARTAGLIA. declare no conflict of interests.
